# Supplementary material for: Common pediatric surgical conditions and associated health-seeking behaviors in Pakistan: An urban and rural comparative assessment
Source: PLOS Glob Public Health. 2024 Sep 12;4(9):e0003327. doi: 10.1371/journal.pgph.0003327 (PMC11392349; doi:10.1371/journal.pgph.0003327)
Supplement: S1 Table — Table A in S1 Table head, Table B in S1 Table eyes, Table C in S1 Table ears, Table D in S1 Table face, Table E in S1 Table neck, Table F in S1 Table chest, Table G in S1 Table back, Table H in S1 Table abdomen, Table I in S1 Table buttocks/groin/genitalia, Table J in S1 Table extremities. (DOCX) [file pgph.0003327.s001.docx]

**S 1: Diagnoses of pictures taken at the study site**

**Table A in S1 text Head**

**Table B in S1 text Eyes**

| **Diagnosis** | **Number of lesions** |
| --- | --- |
| Scar/Keloid | 76 |
| Scarring Alopecia | 47 |
| Hemangioma | 6 |
| Tinea Capitis | 4 |
| Alopecia | 2 |
| Encephalocele | 1 |
| Seborrheic Dermatitis | 1 |

| **Diagnosis** | **Number of lesions** |
| --- | --- |
| Strabismus (Esotropia/Hypertropia) | 43 |
| Scar (Post-traumatic) | 10 |
| Ptosis (Acquired/Congenital) | 9 |
| An-ophthalmus | 3 |
| Corneal opacity | 3 |
| Phthisis bulbi | 2 |
| Nystagmus | 1 |
| Conjunctivitis | 1 |
| Dermoid cyst | 1 |
| Periorbital cellulitis | 1 |

**Table C in S1 text Ears**

| **Diagnosis** | **Number of lesions** |
| --- | --- |
| Discharging ear | 15 |
| Fungal dermatitis | 3 |
| Microtia | 2 |
| Discharging ear with skin excoriation | 1 |
| Pinna dermatitis | 1 |
| Accessory auricle | 1 |

**Table D in S1 text Face**

| **Diagnosis** | **Number of lesions** |
| --- | --- |
| Scar (Post-traumatic)/Post-inflammatory Hypopigmentation | 42 |
| Laceration | 10 |
| Cleft lip/Palate | 6 |
| Post-burn depigmentation | 4 |
| Av malformation | 3 |
| Nasal discharge with excoriation | 2 |
| Tongue tie | 2 |
| Eczema | 2 |
| Atopic dermatitis | 2 |
| Pre-auricular sinus | 2 |
| Post-burn contracture | 1 |
| Angular dermoid | 1 |
| Mucous cyst | 1 |

**Table E in S1 text Neck**

| **Diagnosis** | **Number of lesions** |
| --- | --- |
| Hemangioma | 4 |
| Neck mass/Cyst/Swelling | 4 |
| Scar (Post-traumatic) | 2 |
| Thyroglossal cyst | 2 |
| Torticollis | 1 |
| Burn | 1 |
| Furunculosis | 1 |
| Congenital nevi | 1 |

**Table F in S1 text Chest**

| **Diagnosis** | **Number of lesions** |
| --- | --- |
| Pectus excavatum | 5 |
| Lymphangioma | 3 |
| Ventral Septal Defect | 1 |
| Scar | 1 |
| Clavicle fracture | 1 |

**Table G in S1 text Back**

| **Diagnosis** | **Number of lesions** |
| --- | --- |
| Hemangioma | 4 |
| Soft tissue swelling | 2 |
| Burn | 2 |
| Sprengel deformity | 2 |
| Scar | 1 |
| Spina bifida | 1 |

**Table H in S1 text Abdomen**

| **Diagnosis** | **Number of lesions** |
| --- | --- |
| Umbilical hernia | 22 |
| Scar | 8 |
| Abdominal distension | 6 |
| Umbilical granuloma | 1 |
| Umbilical fistula | 1 |
| Stoma prolapse/Wound dehiscence | 1 |
| Burn | 1 |

**Table I in S1 text Buttocks/Groin/Genitalia**

| **Diagnosis** | **Number of lesions** |
| --- | --- |
| Inguinal hernia | 7 |
| Gluteal abscess | 6 |
| Hydrocele | 2 |
| Burns | 2 |
| Exstrophy bladder | 1 |
| Undescended testes | 1 |

| **Diagnosis** | **Number of lesions** | |
| --- | --- | --- |
|  | **Upper Limb** | **Lower Limb** |
| Leishmaniasis | 4 | 6 |
| Pediculosis | 0 | 1 |
| Atopic dermatitis | 6 | 8 |
| Tinea corporis | 12 | 14 |
| Onychomycosis | 5 | 9 |
| Furunculosis | 5 | 10 |
| Tinea pedis | 0 | 13 |
| Pyoderma gangrenosum | 1 | 3 |
| Vitiligo | 5 | 4 |
| Subungual wart | 5 | 6 |
| Nodule | 3 | 1 |
| Blister | 12 | 7 |
| Eczema | 10 | 3 |
| Keloid | 4 | 6 |
| Deep fungal infection | 11 | 5 |
| Ichthyosis | 5 | 2 |
| Plantar keratoderma | 3 | 1 |
| Post-burn depigmentation | 12 | 6 |
| Femur shaft fracture | 0 | 1 |
| Brachymetatarsia | 0 | 2 |
| Laceration/Scar | 12 | 16 |
| Polydactyly | 7 | 6 |
| Achilles tendinitis | 0 | 4 |
| Cubitus valgus | 1 | 0 |
| Post-polio deformity | 0 | 1 |
| Tibialis posterior tendinitis | 0 | 1 |
| Finger amputation | 1 | 2 |
| Foot ulcer | 0 | 6 |
| Metatarsal head laceration | 0 | 1 |
| Lymphedema | 0 | 2 |
| Genovarum | 0 | 2 |
| Congenital dislocation of knee joint | 0 | 1 |
| Burn contracture | 8 | 2 |
| Clubfoot | 0 | 1 |
| Advance flatfoot deformity | 0 | 1 |
| Gunstock deformity | 1 | 0 |
| Hemimelia | 1 | 0 |
| Paget’s disease | 1 | 0 |
| Nail bed injury | 3 | 4 |
| Plantar wart | 0 | 5 |
| Erb’s palsy | 1 | 0 |
| Volkman ischemic contracture | 1 | 0 |
| Ganglion | 2 | 0 |
| Extensor tendon injury | 0 | 1 |
| Web space laceration | 4 | 2 |
| Postop contracture | 1 | 0 |
| Pulp abscess | 7 | 3 |
| Raynaud’s finger | 1 | 0 |
| Cubitus valgus | 1 | 0 |
| Post supracondylar fracture cubitus varus | 1 | 0 |

**Table J in S1 text Extremities**
