# Supplementary material for: Common pediatric surgical conditions and associated health-seeking behaviors in Pakistan: An urban and rural comparative assessment
Source: PLOS Glob Public Health. 2024 Sep 12;4(9):e0003327. doi: 10.1371/journal.pgph.0003327 (PMC11392349; doi:10.1371/journal.pgph.0003327)
Supplement: Attachment — Submitted filename: Response to Reviewers.docx [file pgph.0003327.s002.docx]

| Reviewer’s comments | Responses |
| --- | --- |
| **REVIEWER 1** | |
| This study asks a good question, what are the most common surgical issues in children in Pakistan, comparing rural and urban in Sindh and Punjab, and what are associated behaviors. This is definitely a research gap, given the population dynamics in Pakistan and healthcare system resource allocations. The language is good, manuscript is written well and is easy to understand. The title is representative of the study, and abstract and conclusions are informative. Good references are provided. | We would like to thank the reviewer for their careful review and thoughtful comments and encouragement |
| This survey may show differences in Sindh and Punjab, especially differences in rural Sindh and rural Punjab. Table 1 indicates the urban vs rural clusters in Sindh and Punjab, but the data is combined after that. Separating information from these two groups may highlight significant differences and add to the information provided in this paper. | We also thought and did that initially, but the numbers are too low for quite a few variables if we divide this by the two provinces and then urban/rural. Also it was not adding to the overall comprehension of the results, hence we focused on presenting this way. Hope this answers your question. |
| The authors find almost half the cases that did not seek care felt it was not needed. Does the survey support this based on the interview and photos reviewed by experts? | The survey identified that most conditions that the caregivers thought did not require care in fact did require surgical care, some acute and some requiring follow-ups. |
| Pakistan has more doctors/1000, can the authors comment on Punjab vs Sindh, and urban vs rural differences in the clusters surveyed? | Thanks, we have now added this information to the discussion section. |
| The discussion regarding care at health centers (tertiary, district via referral systems) (lines 333 to 343) seems out of place based on this survey. As the authors themselves pointed out, Pakistan has more doctors, and there is no significant difference between overall unmet surgical needs in children in urban and rural setting. The authors have not provided any information regarding density of doctors in the surveyed clusters, or an objective assessment of access to hospitals, clinics, and emergency rooms. | Noted and edited accordingly. Thank you. |
| Based on Table 3, most of the children were treated at a healthcare facility (> 80 % in each group), but most received only medical treatment (> 50 % in each group). The authors mention trauma care guidelines. A better discussion about this finding will be helpful. | Thank you for identifying the need to improve this part. We have now edited this section in the discussion. |
| Punjab has a more developed healthcare system compared to other provinces in Pakistan. The study may underrepresent the overall picture, as Baluchistan and KPK findings may be quite different. Did the authors consider this as a limitation of their study? | Thank you so much for the valuable suggestions. They are noted and added to the discussion. |
| Authors noted percentages in the tables, does their analysis show any statistical differences that might be of interest to the reader, especially in Table 3? Appreciate the analysis presented in Table 4 which includes P values. | Thanks for pointing this – we have now added the statistical diff in table 3 |
| **REVIEWER 2** | |
| Thank you for a well-written manuscript. The topic is a very important one and I commend you on taking this up. Please see my comments/suggestions as a peer reviewer. | We would like to thank the reviewer for reviewing the manuscript |
| Line 58 - instead of density could you use "prevalence"? | Noted and addressed. Thank you |
| Line 65 - 'which include "health system...universal health coverage"'. This is not an explicitly mentioned as an SDG. The reference you quote is an interpretation of what is needed to achieve some of the SDGs. I recommend you change the sentence to reflect this. | Noted and addressed. Thank you |
| Line 71 - 'improving the standard of pediatric surgical care'. I recommend that you change it to "improving...surgical care delivery" since that is the gist of your study. Surgical care usually stands for technical surgical skills. | Noted and addressed. Thank you |
| Lines 76-80 - This is good material. Could you add any literature on availability of non-pediatric surgical care? it would be good to position your work in that context. | Noted and addressed. Thank you |
| Line 84 - I suggest changing 'health-seeking' to 'healthcare seeking'. | Noted and addressed. |
| Under Methodology, and elsewhere, please change to active voice. | Noted and addressed. |
| Line 93 - change 'comprised' to 'comprise' | Noted and addressed. |
| Lines 97,98 - A reference to the original (larger) survey is needed here. I would also encourage a 2-3 sentence description of that larger survey. | Noted and addressed. |
| Line 109 - The use of SOSAS concerns me. Why was this chosen? From perusing the site it appears a group involved primarily in providing surgical mission trips- a model that has been criticized for not aiming for native capacity development. Your study is looking at creating long-term strategies for surgical care delivery. Perhaps another tool would have been more useful? Work by Debas [et.al](http://et.al/) (Tanzania), Stewart et al (Ghana) come to mind. - I also recognize that you cannot change a survey tool after the study has been done, and the work you have done is very important; I recommend adding information where SOSAS was used and why you chose it. It would be good to add the limitations and advantages of using SOSAS in the discussion section. | We acknowledge your comment. As this is an area with limited work, we did a search and found SOSAS as a comprehensive validated tool to assess the burden. We recognize the limitations and have listed them in the discussion like relying on recall, in-built definition of surgical conditions, not including chronic conditions etc. The aim of our study is to highlight the surgical burden in children which most stakeholders are not aware of. And, we clearly specify the need for training, resources and capacity building at the local district level. We hope this answers your question. |
| Line 121-126 - It occurs to me that this survey was conducted during the peak of the COVID-19 first waves (with its concomitant social distancing regulations?) . Mentioning this in the discussion section, along with the implications for sampling, and where surgical issues (emergent and non- emergent) where in the respondents radar would be good | This is right and the break in between was due to the lockdown in Pakistan, hence the survey was completed in two phases. This did not hamper the sampling or survey methodology as we stopped the survey during peak COVID when there was lockdown and we started again when things were better and no mass lockdowns in place. |
| Line 130, 131 - Please indicate "To make the survey available on mobile platforms...", it is likely that your readers, as was the case with this reviewer, will not be familiar with the acronyms that you mention here. It would help immensely if you attach references or explain the acronyms. | Noted and addressed. |
| Line 134- 138 - This part really concerned me and left me confused. From the description of the study it appeared that this was a study of perceived need for surgical care; the collection of photos seems to be an attempt at diagnosis- is this appropriate for the study? Was the Ethics Review Board aware of this? If a diagnosis was mode were the patients referred to appropriate care. I understand the challenge of doing a field level survey of surgical conditions and would like a better description of why a 'diagnostic' approach was taken. | We acknowledge the concern and want to assure that all possible ethical principles were taken into account and ethical approvals were sought accordingly. We even had contact numbers of all the households and they were informed where necessary if it was a surgical diagnosis requiring attention, or a part of a larger survey if a child was severely malnourished, anemic, had dental or eyesight issues etc. |
| Line 165 - did you survey children or their parent/guardian? | Both children and their parent/guardians participated in the survey |
| Line 172, 173 - What happens after the diagnosis is made? (see 12 above). Pediatric populations are considered vulnerable populations in most situations. Was the diagnosis communicated back to the patient/family with directions of next steps? | Yes, as mentioned above in the previous response and they were asked to seek healthcare where necessary. |
| Line 170- 247- this section is very elaborate and well written. However, I found myself losing the plot as I read this. With the many tables here, I would encourage you to decrease the written text. | Thanks and we have now significantly rephrased and shortened the text |
| Line 248 - I suggest changing this to "Healthcare Seeking Behavior" | Noted and addressed. |
| Line 300, 301 - It would be good to reflect on the original survey to convey that this was a representative sample, prior to indicating the rural and urban rates. | Thanks, we have now added thos |
| In the discussion section I would recommend, if possible, a framework of looking at healthcare seeking behavior to interpret the results of this study. There are elegant models (Anderson, Gelberg etc) that have looked at this. The Gelberg model would perhaps be a good one to interpret the results with. | Thank you so much for the valuable suggestion. It was noted and added into the discussion section. |
