# Supplementary material for: Common pediatric surgical conditions and associated health-seeking behaviors in Pakistan: An urban and rural comparative assessment
Source: PLOS Glob Public Health. 2024 Sep 12;4(9):e0003327. doi: 10.1371/journal.pgph.0003327 (PMC11392349; doi:10.1371/journal.pgph.0003327)
Supplement: Attachment — Submitted filename: Response to Reviewers.docx [file pgph.0003327.s003.docx]

| Reviewer’s comments | Responses |
| --- | --- |
| **REVIEWER 1** | |
| Reviewer #1: Thank you for revising the manuscript.  I have a few minor comments. | We would like to thank the reviewer for their careful review and thoughtful comments for enhancing the manuscript. |
| Line 33: Recommend change sentence to “This representative cross-sectional household survey conducted on children aged 5-10 years assessed existing surgical diseases and healthcare-seeking behaviors in the two largest provinces (Sindh and Punjab) of Pakistan.” | Noted and addressed. Thank you. |
| Line 41: Recommend change sentence to “13.5% of children surveyed reported a surgical condition, with a similar distribution across urban (13.2%) and rural (13.7) areas.” | Noted and addressed. Thank you. |
| Line 44- Please add a sentence about trauma being the most common underlying reason for the conditions | Noted and addressed. Thank you. |
| Ethics statement: Please add a sentence regarding handling of photos, and communication of diagnosis and treatment recommendations to study participants. This would answer any questions raised in the minds of the readers, similar to concerns raised by a reviewer. | Noted and addressed. Thank you. |
| Table 3- Recommend using “Low Income” and “High Income”, rather than rich and poor. The authors may want to define the income thresholds used for these categories in the methodology section. | Noted and addressed. Thank you. |
| When discussing findings reported in Table 4, use (Table 4) rather than saying “more details in Table 4”. | Noted and addressed. Thank you. |
